# Farmers’ personality traits and credit exclusion: Evidence from rural China

**DOI:** 10.3389/fpsyg.2022.979588

**Published:** 2022-08-26

**Authors:** Yaqun Tian, Yachen Fan, Guangwen He

**Affiliations:** ^1^Rural Development Institute, Chinese Academy of Social Sciences, Beijing, China; ^2^Chinese Academy of Fiscal Sciences, Beijing, China; ^3^College of Economics and Management, China Agricultural University, Beijing, China

**Keywords:** personality traits, farmers, credit exclusion, extroversion factor, agreeableness factor

## Abstract

Unlike existing research from the perspective of financiers or farmers’ financial literacy, this Manuscript investigates the impact of personality traits on Chinese farmers’ credit exclusion using data from 2018 to 2019 of China Agricultural University’s Rural Inclusive Finance Survey. The empirical findings show that farmers’ personality traits significantly affect their credit exclusion. Specifically, conscientiousness and extroversion alleviate the credit exclusion, while agreeableness significantly intensifies the credit exclusion. In addition, the Blinder–Oaxaca decomposition method is used to analyze the contribution of personality traits to each dimension of credit exclusion, and the results of the study show that personality traits mainly affected farmers’ self-exclusion. Therefore, to develop inclusive finance in China, training and improving farmers’ positive personality traits must be fostered.

## Introduction

Rural financial markets in developing countries have serious information asymmetry problems (Giang et al., 2015; Hoff and Stiglitz, 2016), and the credit constraints that farmers face are particularly obvious (Banerjee and Newman, 1993; Zakaria et al., 2019). Therefore, alleviating farmers’ difficulty in acquiring loans has always been the focus of deepening China’s rural inclusive finance system. In 2005, the Chinese government surveyed farmers in 29 provinces, and the results showed that 60.6% of farmers needed loans but only 52.6% of them could obtain funds from banks, which meant that the rate of credit acquisition was about 31.87% (Han et al., 2007). In 2019, China Agricultural University conducted a sample survey on inclusive finance across the country and found that farmers’ rate of credit acquisition was 31.21%, indicating that Chinese farmers still faced serious credit exclusion (He et al., 2018). Over the past decade or so, the Chinese government has required commercial banks to set up more institutions in rural areas and evaluated commercial banks’ agriculture-related loans, which has not only increased the bank branch coverage rate to 97.13% in townships but also caused commercial banks to value rural financial products and services. This has gradually decreased the influence of unreasonable institutional distribution or insufficient financial services on farmers facing credit exclusion (Jayati, 2013). At the same time, a strange phenomenon has arisen in China’s rural financial market: even if credit services are available, many farmers do not obtain or even hate credit services. With sufficient financial supply, are farmers’ traits affecting their financial exclusion? Some scholars have proven that cognitive ability, such as financial literacy (Bernheim and Garrett, 2003; Lusardi, 2012; Agarwal and Mazumder, 2013; Brown et al., 2016; Xu et al., 2022), is an important factor in this phenomenon. So, are there factors aside from cognitive ability?

The rational man hypothesis is the core hypothesis of traditional microeconomics, but individuals are not completely rational when making economic decisions; they are often affected by personality traits and other factors (Heckman, 2006; Kautz et al., 2014; Heckman and Corbin, 2016; Gianpaolo and Peijnenburg, 2019). In the existing research literature, personality traits are generally defined as a series of behavioral habits, or cognitive and affective patterns formed under the influence of genetics and environmental factors (Roberts, 2009). Personality traits are quite stable and unique and can affect individual responses in different situations (Mischel, 1973; Costa and McCrae, 1992; Almlund et al., 2011; Schultz and Schultz, 2016; Salameh et al., 2022), which supports introducing personality traits into economic research to explain individual economic behaviors and derive a personality economics (Heckman, 2011; Li and Zhang, 2015). As the formation of personality traits is influenced by the interaction of innate heredity and acquired environment (Caspi et al., 2005; Heineck and Anger, 2010; Anger and Schnitzlein, 2017), it is easy for farmers to form personality traits that are different from those of urban residents because of their unique living environment in rural China. For example, traditional Chinese farmers are often described as amiable, diligent, and tough (Potter, 1968; Zhou and Li, 2022). However, farmers are also conservative, dependent, and strongly influenced by the small-scale peasant economy and patriarchal family system (Skinner, 1971; Li, 2022). Therefore, this Manuscript demonstrates that if farmers have certain personality traits that enable them to actively connect with financial institutions, establish a receptive attitude, and then form the intention to actively seek credit support from formal financial institutions (Brown and Taylor, 2014), they can obtain formal financial credit services and ultimately, reduce credit exclusion—or not.

The existing research fails to discuss financial exclusion from the perspective of personality traits. Thus, this Manuscript has two contributions: first, it studies the influence of personality traits on credit exclusion in depth and promotes interdisciplinary research in psychology and finance; second, it focuses on Chinese farmers and their distinctive Chinese traits. Because farmers’ issues are a key issue in China’s development, China, as a responsible large country, must ensure that farmers enjoy basic financial rights. Research on this issue will help regulators adjust rural financial policies to better serve rural revitalization.

## Theoretical framework and hypotheses

### Personality traits

Personality traits are relatively stable and are a comprehensive reflection of different psychological characteristics. McAdams (1994) believes that personality characteristics determine the different modes of thinking and behavior among individuals, as well as the unique adjustment modes to the environment. As personality is an abstract concept, how to measure it scientifically is in advance of carrying out relevant research. The “Big Five” personality measurement method is widely used by scholars. It was first proposed by Allport and Odbert (1936), that is, by classifying and summarizing the daily words used by individuals, the main differences of personality characteristics can be measured. And on that basis, Costa and McCrae (1992) classified personality traits into five personality types: conscientiousness, extroversion, openness, agreeableness, and neuroticism, creating the widely accepted “Big Five personality theory.” Conscientiousness reflects an individual’s self-discipline, rationality, and prudence in dealing with others; extroversion describes how enthusiastic, gregarious, talkative, and active an individual is in dealing with others; openness mainly highlights an individual’s curiosity, innovation, and creativity toward new things; agreeableness reflects their altruistic tendencies such as trust, empathy, and obedience to others; and neuroticism emphasizes individual emotional instability or emotional tendencies, and mainly manifests as inner depression, anxiety, poor tolerance, and difficulty in facing setbacks. Further, conscientiousness, extroversion, and openness are considered positive personality traits, while agreeableness and neuroticism are considered negative personality traits (Cheng and Li, 2017).

### Personality traits and credit exclusion

Kempson and Whyley (1999) divided financial exclusion into six dimensions: geographic exclusion, price exclusion, evaluation exclusion, conditional exclusion, marketing exclusion, and self-exclusion. Credit exclusion is a type of financial exclusion that refers to the customer’s inability or unwillingness to obtain credit services (Panigyrakis et al., 2002). Most scholars believe that in rural areas, factors such as the unreasonable distribution of financial institutions (Agarwal and Hauswald, 2010; Dong and Xu, 2012; Dai, 2022), insufficient financial infrastructure (Degryse and Ongena, 2005; Wang et al., 2013), mismatched service supply (Leyshon and Thrift, 1994; Alessandrini et al., 2009; Hollander and Verriest, 2016), and insufficient penetration of financial institutions (Cooper and Zhu, 2018; Cai M. et al., 2020) can easily lead to financial exclusion. Some scholars have analyzed this phenomenon from the perspective of financial literacy and how a low level of education or financial knowledge can lead to self-credit exclusion (Su and Fang, 2016; Zhang and Yin, 2016; Pinjisakikool, 2017). In addition to cognitive abilities, such as financial literacy, a growing body of research has shown that non-cognitive abilities, such as personality traits, are also important components of individual abilities, influencing people’s economic and financial decisions (Becker et al., 2012; Thiel and Thomsen, 2013).

Davey and George (2011) examined the effects of personality traits on financial attitudes and behaviors and it is stated that conscientiousness and extraversion affected their savings and borrowing behaviors more than others. Kubilay and Bayrakdaroglu (2016) examined the personality traits, psychological tendencies and financial risk tolerance of the individual investor. It was stated that there was a significant relationship between the personality traits and psychological tendencies of the investors and the personal characteristics affect the financial risk tolerance. If investors think credit management is risky, they will refuse to lend. Gianpaolo and Kim (2017) found that financial distress and choices are affected by non-cognitive abilities. In a representative panel of households, they found that people in the bottom decile of non-cognitive abilities are five times more likely to experience credit distress compared to those in the top decile. Camelia and Brian (2018) conducted that individuals with high self-efficacy are more likely to take precautions that mitigate adverse financial shocks. They are subsequently less likely to default on financial exclusion. Ozer and Mutlu (2019) deduces that there is a direct relationship between the personality traits and financial behaviors. Among the various elements of personality traits, conscientiousness, agreeableness, and openness to experience were found to be related to financial behaviors.

The deep-rooted clan culture, friendly neighborly culture, and family-based ideology in rural China make it easy for farmers to find guarantors and obtain loans (Gao and Huang, 2019). However, farmers are still largely excluded from acquiring credit. Information collection and processing are important processes in credit decision-making behavior (Choi and Laschever, 2018). However, the credit decision is not the only decision in a family’s economic life (Bortoli et al., 2019). Farmers must also make many decisions about agricultural production and consumption (Giovanni et al., 2017), and each decision requires substantial time and energy (Cobb-Clark et al., 2016; Brooks and Williams, 2021). Therefore, farmers are often unwilling to spend time and energy on researching the family’s credit needs, instead following the “inertia” of previous family fund allocation plans and exhibiting reluctance to obtain help from external financial institutions (Reis, 2006; Dimmock et al., 2016; Dong et al., 2017; Fei, 2017). In addition, when farmers are faced with complicated information, they are often depressed and agitated (Parise and Peijnenburg, 2019), making it difficult for them to sort out and obtain useful information. Without access to sufficient information, household decision-makers cannot choose effectively and actively exclude themselves from credit services. Therefore, we propose:

*Hypothesis 1*: Personality traits affect farmers’ credit exclusion, and mainly affect farmers’ self-exclusion.

Different types of personality traits manifest in differentiated social networks and information transfer abilities, leading to varying degrees of credit exclusion (Borghans et al., 2008; Wang and Qiu, 2011; He et al., 2017; He and Yue, 2021). From the perspective of the social network, the lack of legally qualified collateral is the main reason farmers face credit exclusion (Tian and Fan, 2020), but a developed social network grants farmers sufficient guarantee capacity when applying for formal credit (Vodosek, 2003; Zhao et al., 2010; Zhang et al., 2017; Li et al., 2022). Studies have found that individuals with strong communication motivation and communication skills are more likely to have mature social network relationships (Diener et al., 1984; Li et al., 2018; Zhang and Ji, 2020). Compared with those with negative personalities, farmers with extroverted and open-minded personality traits more strongly prefer social interaction, and farmers with conscientious personalities are more likely to gain others’ trust (Dohmen et al., 2010; Donnelly et al., 2012), so farmers with positive personalities often have broader social networks and find guarantors more easily. Considering information transmission ability, individuals with positive personalities usually have strong transmission abilities and their economic information is more likely to be accurately assessed by financial institutions (van Rooij et al., 2011; Li and Zhang, 2015; Song et al., 2017), while farmers with prominent negative personality traits often find it difficult to express their economic information clearly and tend to convey negative information (Borghans et al., 2008; Oehler et al., 2018), which increases the possibility of banks rejecting their loan applications (Elul et al., 2010). Therefore, we propose:

*Hypothesis 2*: The degree of credit exclusion is related to personality traits. Farmers with positive personalities have a low degree of credit exclusion, and those with negative personalities have a high degree of credit exclusion.

## Materials and methods

The data in this article come from the 2018 and 2019 China Rural Financial Inclusion Survey conducted by the School of Economics and Management of the China Agricultural University. The survey uses stratified random sampling. According to China’s administrative divisions, three provinces are randomly selected from the eastern, central, and western regions. Based on per capita gross domestic product (GDP), each province selects three counties with high, medium, and low levels of economic development. The investigation team selected Shouguang County, Gaomi County, and Anqiu County in Shandong Province in the eastern region, Fangcheng County, Mengjin County, and Tangyin County in Henan Province in the central region, and Wuchuan County, Dafang County, and Fuquan County in Guizhou Province in the western region. Each county then selected three towns according to their level of economic development, and then randomly selected two villages in each town and 20–30 sample farmers in each sample village. In 2018 and 2019, the research team issued a total of 3,706 questionnaires and received 3,215 valid questionnaires, with an effectiveness rate of 86.75% (Table 1).

**TABLE 1:** Statistical table of the distribution of survey questionnaires.

Province	County	Number of received	Number of issued	Efficiency (%)
Shandong	Shouguang	381	439	86.79
	Gaomi	356	427	83.37
	Anqiu	343	408	84.07
Henan	Fangcheng	337	396	85.10
	Mengjin	350	424	82.55
	Tangyin	410	485	84.54
Guangxi	Wuchuan	345	370	93.24
	Fuquan	351	375	93.60
	Dafang	342	384	89.06
Total		3,215	3,706	86.75

### Measurement of the variables

#### Dependent variable

This Manuscript draws on the discrete element method (DEM) method of previous research on credit rationing (Devlin, 2005), and mainly judges whether farmers are excluded from credit by the following three questions (Table 2).

**TABLE 2:** List of credit exclusion questions.

	Question	Options
Q1	Have you obtained the following loans from a bank?	(1) Loans other than education loans; (2) Education loans; (3) Never applied; and (4) Applied but was rejected.
Q2	If you chose “3. Never applied” in Q1, what is the reason?	(1) No need; (2) Do not know how to apply; (3) No collateral; (4) No guarantor; (5) Do not know bank staff; (6) Can borrow money from other places; (7) Applying for a loan takes a long time; (8) High interest rate; (9) Short loan term; and (10) Worry about repayment
Q3	If you chose “4. Applied but was rejected” in Q1, what is the reason?	(1) No collateral or guarantor; (2) Do not know bank staff; (3) Low income; (4) Unpaid loans; and (5) Others

If the respondent chose “3–Never applied” in Q1, and the answer to Q2 is not “1–No need,” the respondent has been excluded from credit; likewise, if the respondent chose “4–Applied but was rejected,” we argue that the respondent is subject to credit exclusion. Credit exclusion is recorded as Exclu_credit = 1; if other answers were selected, the respondent was not subject to credit exclusion, which is recorded as Exclu_credit = 0.

To analyze credit exclusion in depth, this Manuscript divides the types of credit exclusion into three categories: (1) Self-exclusion, in which farmers believe that banks will not lend them money or habitually borrow money from relatives and friends without trying to apply for loans; (2) Conditional exclusion, in which farmers are excluded from credit activities due to high loan conditions, such as a lack of collateral or guarantors or the high risk of loan projects; (3) Price exclusion, in which farmers do not apply to banks because of high loan interest, time cost, or bank fees. The specific division method is shown in Table 3.

**TABLE 3:** Credit exclusion classification.

Credit exclusion classification	Q1: Have you obtained the following loans from a bank? (1) Loans other than education loans; (2) Education loans; (3) Never applied; and (4) Applied but was rejected
Not rejected for by credit	(1) Loans other than education loans; (2) Education loans; and (4) Applied but was rejected [(4) Unpaid loans]
Self-exclusion	(3) Never applied [(1) No need; (2) Do not know how to apply; (6) Can borrow money from other places; and (10) Worry about repayment]
Conditional exclusion	(3) Never applied [(3) No collateral; (4) No guarantor; (7) Applying for a loan takes a long time; (9) Short loan term] (4) Applied but was rejected [(3) Low income]
Price exclusion	(3) Never applied [(8) High interest rate]

[] indicates the reason for the choice. Key Independent Variable.

According to the Big Five personality taxonomy (Costa and McCrae, 1992), everyone has five personality traits, namely conscientiousness (con), extroversion (ext), openness (ope), agreeableness (agr), and neuroticism (neu). Based on this theory, a series of questions were set in the survey to identify each personality trait, and each question was assigned a corresponding score. Considering the many sub-problems involved in each personality trait, a factor analysis method was adopted to reduce the dimensionality of the sub-problems and select factors with Kaiser–Meyer–Olkin (KMO) values greater than 0.7 to align with the five personality traits, the extracted factors were named the conscientiousness factor, extroversion factor, openness factor, agreeableness factor, and neuroticism factor. Conscientiousness, extroversion, and openness were defined as positive personality traits, while agreeableness and neuroticism were defined as negative personality traits, as shown in Table 4.

**TABLE 4:** Big Five personality characteristic classification and corresponding questions.

	Big Five personality traits	Dimensions	Corresponding questions
Positive personality traits	Conscientiousness	Organization	Degree of neatness of respondents’ dress; 1 (very poor)–5 (very good)
			Degree of tidiness inside the home; 1 (very poor)–5 (very good)
		Striving and aggression	Do you think “the sense of achievement in life” is important? (1) very unimportant; (2) unimportant; (3) general; (4) important; and (5) very important
			Do you think hard work will pay off? (1) strongly disagree; (2) disagree; (3) general; (4) agree; and (5) strongly agree
		Prudence	Respondent’s level of doubt about the survey. (1) very doubtful; (2 doubtful; (3) neutral; (4) trusting; and (5) very trusting
	Extroversion	Ability to handle interpersonal relationships	Respondent’s level of hospitality; 1 (very low)–5 (very high)
		Gregariousness	Do you think “not being alone” is important? (1) very unimportant; (2) unimportant; (3) general; (4) important; and (5) very important
		Positive attitude towards life	Do you think “having fun in life” is important? (1) very unimportant; (2) unimportant; (3) general; (4) important; and (5) very important
	Openness	Attention to new things	Respondents’ interest in the survey; 1 (very low)–5 (very high)
			Do you usually pay attention to entrepreneurial information? (1) very unconcerned; (2) unconcerned; (3) general; (4) concerned; and (5) very concerned
			Do you usually pay attention to the government’s policies on entrepreneurship? (1) very unconcerned; (2) unconcerned; (3) general; (4) concerned; and (5) very concerned
			Do you usually pay attention to entrepreneurship training or entrepreneurship knowledge lectures? (1) very unconcerned; (2) unconcerned; (3) general; (4) concerned; and (5) very concerned
			Do you understand the policy of supporting farmers’ self-employment? (1) Do not understand very much; (2) Do not understand; (3) general; (4) understand; and (5) understand very well
		Disagreement with traditional values	Do you think it is important to “must have a son to pass on the lineage”? (1) Very important; (2) important; (3) general; (4) unimportant; and (5) very unimportant;
Negative personality traits	Agreeableness	Trust	Do you trust strangers? (1) very distrust; (2) distrust; (3) general; (4) trust; and (5) very trust
		Altruism	Do you think “not being hated” is important? (1) very important; (2) important; (3) general; (4) more important; and (5) very important
		Obedience	Respondent’s degree of cooperation with the survey; 1 (very low)–5 (very high)
	Neuroticism	Anxiety	Have you felt “nervous” in the past week? (1) Hardly; (2) Sometimes (1–2 days); (3) Often (3–4 days); and (4) Most of the time (5–7 days)
			Have you felt “difficult to stay calm” over the past week? (1) Hardly; (2) Sometimes (1–2 days); (3) Often (3–4 days); and (4) Most of the time (5–7 days)
		Depression or psychological fragility	Have you experienced “I feel depressed, even with help from family and friends” in the past week? (1) Hardly; (2) Sometimes (1–2 days); (3) Often (3–4 days); and (4) Most of the time (5–7 days)
			Have you felt “hopeless for the future” this past week? (1) Hardly (less than a day); (2) Sometimes (1–2 days); (3) Often (3–4 days); and (4) Most of the time (5–7 days)
			Have you felt like “I do not have the energy to do anything” this past week? (1) Hardly (less than a day); (2) Sometimes (1–2 days); (3) Often (3–4 days); and (4) Most of the time (5–7 days)
			Have you felt “life does not make sense” in the past week? (1) Hardly (less than a day); (2) Sometimes (1–2 days); (3) Often (3–4 days); and (4) Most of the time (5–7 days)

#### Control variable

After reviewing the existing literature, we selected the control variables that jointly affect the credit exclusion and personality traits of farmers according to three aspects: individual characteristics variables, household characteristics variables, and regional development variables (Cho, 2016; Han and Chen, 2019; Cai Q. F. et al., 2020; Guan et al., 2020). Among these, personal characteristic variables include gender (Niu et al., 2022), age (Liao et al., 2019), education level (Zhu et al., 2021), marital status (Shen et al., 2022) and financial literacy (Yin and Zhang, 2020). The identification question for financial literacy is “Suppose you have 100 yuan in savings at 2% interest rate for 5 years, how much money will you have in your account after 5 years?” If the farmer answers correctly, Financial_literacy = 1, otherwise, Financial_literacy = 0. Household characteristics variables include the number of family members (Wang and He, 2020), the number of family members in the labor force (Fan et al., 2022), whether there are village officials in the family (Xue, 2022), whether there are college students in the family (Zhang and Li, 2022), and family income (Tian and Zhang, 2022). Regional development variables include per capita income (Jin et al., 2022) and the county’s financial market structure (Wang and He, 2020; Wang and Bei, 2022). The descriptive statistics of variables is shown in Table 5.

**TABLE 5:** Descriptive statistics.

Variable			Mean	Std. dev.	Observations
Dependent variable	Percentage of credit exclusion		0.337	0.463	3215
	Percentage of self-exclusion		0.109	0.297	3215
	Percentage of conditional exclusion		0.078	0.362	3215
	Percentage of price exclusion		0.032	0.225	3215
Independent variable	Positive personality traits	Conscientiousness factor	3.94	0.915	3215
		Extroversion factor	5.281	0.874	3215
		Openness factor	2.172	0.536	3215
	Negative personality traits	Agreeableness factor	3.578	1.618	3215
		Neuroticism factor	1.366	0.523	3215
Control variables	Individual characteristics variables	Gender (1 = male; 0 = female)	0.53	0.495	3215
		Age (years)	48.875	12.541	3215
		Education (years)	7.222	3.532	3215
		Marriage (1 = yes; 0 = no)	1.132	0.302	3215
		Financial Literacy (1 = yes; 0 = no)	0.118	0.283	3215
	Household characteristics variables	Number of family members	4.379	1.917	3215
		Labor force	2.643	1.163	3215
		Village officials (1 = yes; 0 = no)	0.043	0.198	3215
		College students (1 = yes; 0 = no)	0.238	0.672	3215
		Family income (Ten thousand yuan)	6.514	5.71	3215
	Regional development variables	GDP per capita (Ten thousand yuan)	4.831	2.102	3215
		Financial market structure	0.064	0.049	3215

### Estimation strategy

This Manuscript mainly reports on the influence of farmers’ personality traits on the degree of credit exclusion they experience and further explores the influence of personality traits on different dimensions of farmers’ credit exclusion. First, Probit Model is used to estimate whether farmers’ credit exclusion is related to their personality traits. Then, a follow-up regression is conducted to test whether the personality traits of sample farmers lead to self-exclusion, conditional and evaluation exclusion, or price exclusion among farmers under credit exclusion. The model is as follows:


$$\mathrm{Pr}\text{exclu}\,\_\,\mathrm{credit}=\alpha_{0}+\alpha_{1}\,C\,o\,n\,s\,c\,i\,e\,n\,t\,i\,o\,u\,s\,n\,e\,s\,s\,\_\,F\,a\,c\,t\,o\,r_{i}+\alpha_{2}E\,x\,t\,r\,o\,v\,e\,r\,s\,i\,o\,n\,\_\,F\,a\,c\,t\,o\,r_{i}+\alpha_{3}\,O\,p\,e\,n\,n\,e\,s\,s\,\_\,F\,a\,c\,t\,o\,r_{i}+\alpha_{4}\,A\,g\,r\,e\,e\,a\,b\,l\,e\,n\,e\,s\,s$$

(1) $$\_\,F\,a\,c\,t\,o\,r_{i}+\alpha_{5}\,N\,e\,u\,r\,o\,t\,i\,c\,i\,s\,m\,\_\,F\,a\,c\,t\,o\,r_{i}+\alpha_{i}\,c\,o\,n\,t\,r\,o\,l\,s_{i}+\eta_{i}$$

Where *exclu*_*credit* is a dummy variable; when exclu_credit=1, the farmer is subject to credit constraints, otherwise, exclu_credit=0. The variables of the farmers’ personality traits are the *Conscientiousness*_*Factor*_*i*_, *Extroversion*_*Factor*_*i*_, *Openness*_*Factor*_*i*_, *Agreeableness*_*Factor*_*i*_, and *Neuroticism*_*Factor*_*i*_; *controls*_*i*_ presents a series of control variables; and η_*i*_ is the random error term.

In cases where farmers experience credit exclusion, this Manuscript further studies which personality characteristics affect self-exclusion, conditional exclusion, and price exclusion, and the model is:


$$\text{E}\,{\text{exclu}}_{i}|{\mathrm{exclu}}_{c}\,\mathrm{redit}=1+\beta_{0}+\beta_{1}\,C\,o\,n\,s\,c\,i\,e\,n\,t\,i\,o\,u\,s\,n\,e\,s\,s_{F}\,a\,c\,t\,o\,r_{i}+\beta_{2}\,E\,x\,t\,r\,o\,v\,e\,r\,s\,i\,o\,n_{F}\,a\,c\,t\,o\,r_{i}+\beta_{3}\,O\,p\,e\,n\,n\,e\,s\,s_{F}\,a\,c\,t\,o\,r_{i}+\beta_{4}$$

(2) $$A\,g\,r\,e\,e\,a\,b\,l\,e\,n\,e\,s\,s_{F}\,a\,c\,t\,o\,r_{i}+\beta_{5}\,N\,e\,u\,r\,o\,t\,i\,c\,i\,s\,m_{F}\,a\,c\,t\,o\,r_{i}+\beta_{i}\,c\,o\,n\,t\,r\,o\,l\,s_{i}+\varepsilon_{i}$$

Where exclu_*i*_ represents the dummy variable of self-exclusion, conditional exclusion, or price exclusion.

## Results

### Descriptive statistics

According to the statistical results of the surveyed sample, one-third of the sample farmers are excluded from credit. Of the total sample, 10.9% are self-excluded, 7.8% are excluded conditionally, 3.2% are subject to price exclusion, and the remaining farmers experiencing exclusion did not explain the reason. In addition, the statistical results reflect the universal traits of rural families in China: (1) families usually have four members, with 2–3 workers and a total annual family income of about 65,100 yuan; (2) Household heads have received 7 years of education on average and have not completed the compulsory education period stipulated in China; (3) About 25% of families include college students, and only 4.3% of families include village officials.

### Baseline results

Positive personality factors are negatively correlated with farmers’ credit exclusion. That is, the more positive a farmer’s personality is, the less likely they are to be rejected for credit. Of the negative personality traits, agreeableness factors significantly increase the degree of credit exclusion that farmers experience, indicating that the more agreeableness they exhibit, the greater the degree of credit exclusion they will experience. Neuroticism has no significant effect on credit rejection.

The sub-dimension empirical results show that (1) The regression results for self-exclusion show that the higher the conscientiousness and extroversion factors in the positive personality traits, the lower the probability of farmers being self-excluded; the higher the agreeableness and neuroticism factors in the negative personality traits, the higher the probability of self-rejection. (2) The regression results of conditional rejection show that the coefficient of the extroversion factor is significantly negative, indicating that the more extroversion a farmer has, the less likely they will be subject to conditional rejection. The coefficients of both the agreeableness and neuroticism factors are positive, indicating that negative personality traits increase the conditional rejection of farmers to some extent. (3) The regression results of price exclusion show that the extroversion, openness, and agreeableness factors significantly affect price exclusion, which means that higher extroversion and openness in farmers can reduce the price exclusion they experience, while higher agreeableness increases the possibility of price exclusion.

To explore the influence of farmers’ personality traits on self-exclusion, conditional exclusion, and price exclusion, the Blinder–Oaxaca decomposition method was adopted to analyze personality traits’ contributions to each dimension of credit exclusion by constructing combinations of different personality traits, referring to Wu’s (2019) study. The Blinder–Oaxaca decomposition method is often used to analyze the contribution of factors between groups, which reflects the idea of counterfactual analysis (Fairlie, 2005). The regression results in Table 6 show that the extroversion and agreeableness factors are the primary factors affecting credit exclusion. Therefore, we constructed two combinations of different levels of extroversion and agreeableness. In this study, based on Ling et al. (2017), the farmers in the bottom third of the extroversion factor scores and the top third of the agreeableness factor scores are regarded as the low-level group (control group); these farmers face high credit exclusion. The farmers in the top third of the extroversion factor scores and the bottom third of the agreeableness factor scores are regarded as the high-level group (experimental group) and face lower credit exclusion. On this basis, the credit exclusion functions of the high-level and low-level groups are established:

**TABLE 6:** Baseline regressions.

Variable	Credit exclusion	Self-exclusion	Conditional exclusion	Price exclusion
Conscientiousness factor	−0.073*	−0.215***	–0.060	–0.013
	(0.036)	(0.059)	(0.051)	(0.064)
Extroversion factor	−0.182***	−0.102**	−0.031**	–0.044
	(0.059)	(0.046)	(0.012)	(0.027)
Openness factor	−0.078*	–0.043	–0.047	−0.069*
	(0.045)	(0.055)	(0.045)	(0.041)
Agreeableness factor	0.154***	0.078*	0.052*	0.041
	(0.055)	(0.045)	(0.029)	(−0.038)
Neuroticism factor	0.057	0.035***	0.114***	0.027
	(0.039)	(0.012)	(0.023)	(0.016)
Gender	−0.033*	–0.057	–0.044	–0.025
	(0.019)	(0.048)	(0.030)	(0.031)
Age	0.015*	0.018	0.0059**	0.019
	(0.008)	(0.011)	(0.002)	(0.014)
Education	0.039***	0.001	0.013**	0.044
	(0.007)	(0.001)	(0.006)	(0.034)
Marriage	0.0199	0.0267	0.0204	0.0138
	(0.053)	(0.077)	(0.050)	(0.031)
Financial literacy	0.025	–0.029	0.033	–0.028
	(0.033)	(0.044)	(0.042)	(0.020)
Number of family members	0.029	0.057	0.042	0.051
	(0.032)	(0.036)	(0.028)	(0.042)
Labor force	−0.025***	−0.017***	–0.023	–0.029
	(0.006)	(0.005)	(0.022)	(0.020)
Village officials	−0.061*	–0.015	−0.074*	–0.021
	(0.036)	(0.012)	(0.043)	(0.014)
College student	–0.042	–0.021	−0.049*	–0.024
	(0.031)	(0.026)	(0.029)	(0.025)
Family income	−0.079*	–0.042	–0.016	–0.046
	(0.047)	(0.031)	(0.012)	(0.029)
GDP per capita	0.049	0.023	0.058	0.014
	(0.037)	(0.051)	(0.044)	(0.051)
Financial market structure	0.043*	0.009	0.045	0.027
	(0.024)	(0.012)	(0.042)	(0.028)
Constant	3.923***	2.539	1.176	2.307***
	(0.617)	(−2.399)	(0.906)	(0.451)

Robust standard errors in parentheses.

*p < 0.10, **p < 0.05, and ***p < 0.01.


(3) $${\text{exclu\_credit\_high}}_{m}=\gamma_{0}+\gamma_{m}\,p\,e\,r\,s\,o\,n\,a\,l\,i\,t\,i\,e\,s_{m}+\gamma_{i}\,c\,o\,n\,t\,r\,o\,l\,s_{i}+\varepsilon_{i}$$

(4) $${\text{exclu\_credit\_low}}_{w}=\delta_{0}+\delta_{w}\,p\,e\,r\,s\,o\,n\,a\,l\,i\,t\,i\,e\,s_{w}+\delta_{i}\,c\,o\,n\,t\,r\,o\,l\,s_{i}+\varepsilon_{i}$$

Where exclu_credit_high_*m*_ and exclu_credit_low_*w*_ are credit exclusions in the high-level and low-level groups, respectively; *personalities*_*m*_ and *personalities*_*w*_ correspond to the personality characteristics of each group, *controls*_*i*_ is a series of control variables, and ε_*i*_ is the random error term.

The factors affecting farmers’ credit exclusion in the two groups were estimated, and the differences in regression coefficients between the two groups were decomposed according to the Oaxaca-Blinder decomposition method as follows:


(5) $$\text{E}\,\left(\text{exclu\_credit\_high}\right)-\text{E}\,\left(\text{exclu\_credit\_low}\right)=\left(\text{E}\,\left(p\,e\,r\,s\,o\,n\,a\,l\,i\,t\,i\,e\,s_{m}\right)-\text{E}\,\left(p\,e\,r\,s\,o\,n\,a\,l\,i\,t\,i\,e\,s_{w}\right)\right)\delta_{w}+\left(\gamma_{m}-\delta_{w}\right)\,E\,\left(p\,e\,r\,s\,o\,n\,a\,l\,i\,t\,i\,e\,s_{m}\right)+\varepsilon_{i}$$

Where E(*exclu*_*credit*_*high*)−E(*exclu*_*credit*_*low*) is the difference between the eigenvalues of the high- and low-level groups, generally referred to as the interpretable part. (γ_*m*_−δ_*w*_)*E*(*personalities*_*m*_) is the uninterpretable part. During data analysis, due to the difference in sample size between the two groups, we randomly selected samples from the larger group to match the size of the smaller group, and randomly sampled them 100 times.

The regression results in Table 7 show the significant differences in the influence of personality traits on credit exclusion. Among the factors affecting credit exclusion, personality traits significantly affect self-exclusion, accounting for 83.78% of self-exclusion, but have no significant impact on conditional or price exclusion. This implies that personality traits affect credit exclusion mainly by affecting self-exclusion. Specifically, the extroversion and agreeableness factors primarily affect the overall degree of credit exclusion by influencing self-exclusion, which is consistent with the conclusions Table 6 displays. The farmers with obvious extroverted personalities usually have strong social communication abilities, which helps them obtain the support of guarantors. Conversely, farmers with obvious agreeable personality traits are easily bound by traditional ideas and lack subjective judgment abilities. This may explain why a low score for the extroversion factor and a high score for the agreeableness factor predicts that farmers are more likely to think that banks will not provide them with loans, resulting in credit self-exclusion.

**TABLE 7:** Contribution decomposition.

	Self-exclusion		Conditional exclusion		Price exclusion	
	Coeff	Contribution rate (%)	Coeff	Contribution rate (%)	Coeff	Contribution rate (%)
Differences between groups	0.037*	100.00	0.029	100.00	0.001	100.00
interpretable part	0.031	83.78	0.011	37.93	0.00032	32.00
uninterpretable part	0.006	16.22	0.018	62.07	0.00068	68.00

Robust standard errors in parentheses, *p < 0.10, **p < 0.05, and ***p < 0.01.

### Robustness checks and endogeneity

To demonstrate the robustness of the results, extreme income values are excluded, as people with extremely low incomes are more likely to be excluded from credit, and people with high incomes may be able to meet their spending needs without borrowing. Specifically, the Winsorize method was used to eliminate the highest and lowest 5% of the high- and low-income samples. Robust results show that positive personality traits can significantly reduce farmers’ credit exclusion, while negative personality traits can significantly increase farmers’ credit exclusion.

In addition, the credit exclusion data may have endogeneity problems due to omitted variables, measurement errors or mutual causation. This Manuscript uses an instrumental variable method to solve endogeneity problems. Referring to the method Bucher-Koenen and Lusardi (2011) used, the average of the personality scores of other farmers in a village was adopted as the instrumental variable of the interviewees’ personality traits. The average level of other farmers’ personality traits in the village affected the interviewees’ personality traits (Araujo et al., 2011), which met the requirement to correlate instrumental variables. However, the interviewees’ personality traits could hardly affect the overall level of the village’s personality traits, which also met the exogenous requirements of instrumental variables. The Kleibergen-Paap rk LM values show a strong rejection of the unidentifiable null hypothesis. The Cragg–Donald Wald F values are greater than 10, indicating that the null hypothesis of “weak instrumental variables” is rejected, so the instrumental variables are valid. At the same time, the regression conclusion remains stable (Table 8).

**TABLE 8:** Robustness and endogeneity test regression results.

Variable	Robustness test		Endogeneity test (IV-Probit)	
	Credit exclusion	Self-exclusion	Conditional exclusion	Price exclusion
Conscientiousness factor	–0.077	−0.211***	−0.089*	–0.200
	(0.048)	(0.063)	(0.046)	(0.129)
Extroversion factor	−0.153**	−0.112***	−0.091*	−0.129**
	(0.066)	(0.047)	(0.054)	(0.065)
Openness factor	−0.076*	–0.034	–0.069	–0.029
	(0.043)	(0.055)	(0.050)	(0.054)
Agreeableness factor	0.098*	0.081*	0.158**	0.115
	(0.055)	(0.048)	(0.065)	(0.148)
Neuroticism factor	0.051	0.040*	0.059	0.091
	(0.032)	(0.022)	(0.044)	(0.067)
Gender	–0.038	–0.053	−0.062***	–0.114
	(0.023)	(0.047)	(0.022)	(0.092)
Age	0.016	0.021	0.018**	0.032***
	(0.012)	(0.013)	(0.007)	(0.009)
Education	0.024**	0.001	0.021	0.001
	(0.011)	(0.001)	(0.016)	(0.001)
Marriage	0.021	0.029	0.058	0.030
	(0.049)	(0.074)	(0.052)	(0.076)
Financial literacy	0.0031	0.017	0.035	0.008
	(0.032)	(0.044)	(0.029)	(0.019)
Number of family members	0.022	0.061*	0.035	0.091**
	(0.031)	(0.036)	(0.042)	(0.047)
Labor force	–0.025	−0.026*	−0.044***	–0.004
	(0.019)	(0.014)	(0.015)	(0.005)
Village officials	−0.060***	–0.017	–0.094	–0.131
	(0.025)	(0.012)	(0.102)	(0.158)
College students	–0.032	–0.009	–0.035	–0.022
	(0.033)	(0.022)	(0.045)	(0.019)
Family income	−0.076*	–0.041	−0.077*	–0.045
	(0.047)	(0.032)	(0.044)	(0.034)
GDP per capita	0.052	0.023	0.035	0.019
	(0.037)	(0.059)	(0.041)	(0.031)
Financial market structure	0.038	0.011	0.043	0.009
	(0.024)	(0.012)	(0.029)	(0.012)
Constant	3.615***	1.749***	1.206	2.074**
	(0.607)	(0.509)	(1.742)	(1.052)
Kleibergen-Paap rk LM value			20.59***	21.07***
Cragg–Donald Wald F values			48.39***	51.54***

Robust standard errors in parentheses.

*p < 0.10, **p < 0.05, and ***p < 0.01.

### Mechanism verification

Theoretical analysis suggests that social network and information transmission ability are the mechanisms of personality traits affecting credit exclusion. This Manuscript constructs the following equation to verify whether these mechanisms are valid, referring to Dai’s (2022) study. Among them, the social network identification question is “ If you are sick and in need of money, how many people can you turn to?” The more people you can turn to, the wider your social network. According to the survey, farmers who are proficient in Mandarin generally have strong information transmission ability. Therefore, this Manuscript takes “mandarin proficiency of interviewees (1 very poor–5 very good)” as the identification problem of information transmission ability. The regression results demonstrated the existence of a mechanism of action (Table 9).

**TABLE 9:** Mechanism test regression results test regression results.

Variable	Social_ Network	Infor_transf_Ability	Credit exclusion
Conscientiousness factor	0.041	0.116*	–0.069
	(0.032)	(0.065)	(0.226)
Extroversion factor	0.162***	0.108**	−0.127**
	(0.053)	(0.046)	(0.060)
Openness factor	0.048*	0.021	0.073
	(0.029)	(0.044)	(0.049)
Agreeableness factor	0.092	–0.052	0.136**
	(0.063)	(0.048)	(0.055)
Neuroticism factor	−0.058*	0.027	0.051
	(0.032)	(0.056)	(0.038)
Social_ Network			−0.070*
			(0.042)
Infor_transf_Ability			−0.054*
			(0.033)
Gender	0.029	0.035	–0.052
	(0.030)	(0.047)	(0.049)
Age	0.006	0.015	0.020*
	(0.008)	(0.017)	(0.012)
Education	0.041***	0.052***	0.034***
	(0.007)	(0.016)	(0.005)
Marriage	0.0195	0.0272	0.0201
	(0.051)	(0.063)	(0.066)
Financial literacy	0.036**	0.0041*	0.022
	(0.019)	(0.023)	(0.036)
Number of family members	0.018***	0.058	0.027
	(0.006)	(0.036)	(0.033)
Labor force	0.125***	0.036	−0.019**
	(0.039)	(0.032)	(0.010)
Village officials	1.201***	0.018	−0.065*
	(0.063)	(0.015)	(0.037)
College student	0.044	0.021	–0.044
	(0.031)	(0.026)	(0.035)
Family income	0.080**	0.053	−0.081*
	(0.047)	(0.050)	(0.049)
GDP per capita	0.439	0.196	0.045
	(0.493)	(0.201)	(0.037)
Financial market structure	0.041	0.027	0.069***
	(0.034)	(0.031)	(0.025)
Constant	3.528***	2.169	2.377***
	(0.622)	(1.425)	(0.631)

Robust standard errors in parentheses.

*p < 0.10, **p < 0.05, and ***p < 0.01.

$$S\,o\,c\,i\,a\,l\,\_\,N\,e\,t\,w\,o\,r\,k=\gamma_{0}+\gamma_{1}\,C\,o\,n\,s\,c\,i\,e\,n\,t\,i\,o\,u\,s\,n\,e\,s\,s\,\_\,F\,a\,c\,t\,o\,r_{i}+\gamma_{2}$$

$$E\,x\,t\,r\,o\,v\,e\,r\,s\,i\,o\,n\,\_\,F\,a\,c\,t\,o\,r_{i}+\gamma_{3}\,O\,p\,e\,n\,n\,e\,s\,s\,\_\,F\,a\,c\,t\,o\,r_{i}+\gamma_{4}$$

(6) $$A\,g\,r\,e\,e\,a\,b\,l\,e\,n\,e\,s\,s\,\_\,F\,a\,c\,t\,o\,r_{i}+\gamma_{5}\,N\,e\,u\,r\,o\,t\,i\,c\,i\,s\,m\,\_\,F\,a\,c\,t\,o\,r_{i}+\gamma_{i}\,c\,o\,n\,t\,r\,o\,l\,s_{i}+\eta_{i}$$

$$I\,n\,f\,o\,r\,\_\,t\,r\,a\,n\,s\,f\,\_\,A\,b\,i\,l\,i\,t\,y=\delta_{0}+\delta_{1}\,C\,o\,n\,s\,c\,i\,e\,n\,t\,i\,o\,u\,s\,n\,e\,s\,s\,\_\,F\,a\,c\,t\,o\,r_{i}+\delta_{2}$$

$$E\,x\,t\,r\,o\,v\,e\,r\,s\,i\,o\,n\,\_\,F\,a\,c\,t\,o\,r_{i}+\delta_{3}\,O\,p\,e\,n\,n\,e\,s\,s\,\_\,F\,a\,c\,t\,o\,r_{i}+\delta_{4}$$

(7) $$A\,g\,r\,e\,e\,a\,b\,l\,e\,n\,e\,s\,s\,\_\,F\,a\,c\,t\,o\,r_{i}+\delta_{5}\,N\,e\,u\,r\,o\,t\,i\,c\,i\,s\,m\,\_\,F\,a\,c\,t\,o\,r_{i}+\delta_{i}\,c\,o\,n\,t\,r\,o\,l\,s_{i}+\eta_{i}$$

$$\mathrm{Pr}\text{exclu\_credit}=\delta_{0}+\delta_{1}\,C\,o\,n\,s\,c\,i\,e\,n\,t\,i\,o\,u\,s\,n\,e\,s\,s\,\_\,F\,a\,c\,t\,o\,r_{i}+\delta_{2}$$

$$E\,x\,t\,r\,o\,v\,e\,r\,s\,i\,o\,n\,\_\,F\,a\,c\,t\,o\,r_{i}+\delta_{3}\,O\,p\,e\,n\,n\,e\,s\,s\,\_\,F\,a\,c\,t\,o\,r_{i}+\delta_{4}$$

$$A\,g\,r\,e\,e\,a\,b\,l\,e\,n\,e\,s\,s\,\_\,F\,a\,c\,t\,o\,r_{i}+\delta_{5}\,N\,e\,u\,r\,o\,t\,i\,c\,i\,s\,m\,\_\,F\,a\,c\,t\,o\,r_{i}+\delta_{6}$$

(8) $$S\,o\,c\,i\,a\,l\,\_\,N\,e\,t\,w\,o\,r\,k+\delta_{7}\,I\,n\,f\,o\,r\,\_\,t\,r\,a\,n\,s\,f\,\_\,A\,b\,i\,l\,i\,t\,y+\delta_{i}\,c\,o\,n\,t\,r\,o\,l\,s_{i}+\eta_{i}$$

## Discussion

### Theoretical implications

In this study, the effect of farmer’s personality traits on credit exclusion is analyzed by regression analysis of the data obtained from the 2018 and 2019 China Rural Financial Inclusion Survey conducted by the School of Economics and Management of the China Agricultural University. According to the findings of the analysis, the personality dimensions of conscientiousness, extroversion and openness have a statistically significant effect on the credit exclusion of farmers. However, agreeableness and neuroticism have no significant or negative effect on framer’s credit exclusion. This result is consistent with the results of many studies (Davey and George, 2011; Kubilay and Bayrakdaroglu, 2016; Camelia and Brian, 2018) in the literature. In addition, this Manuscript has some different findings: firstly, the research object of this Manuscript is “vulnerable or marginalized groups”, while some scholars study “high-quality customers”, such as Gianpaolo and Kim (2017) and Ozer and Mutlu (2019). Secondly, different from the general analysis of Camelia and Brian (2018), this Manuscript subdivides credit exclusion into three dimensions, deeply studies the influence of personality traits on credit exclusion of different dimensions, and then finds that personality traits mainly affect self-exclusion in credit exclusion. Thirdly, the significance of this Manuscript is to promote the personality education of farmers and promote their overall development. In contrast, the research of Ozer and Mutlu (2019) focuses on how to guide financial institutions to match user needs according to user personality traits.

### Practical implications

This Manuscript has realistic significance. In China, the realization of inclusive finance requires the joint efforts of both clients and suppliers. From the perspective of the supply side, it is very important to increase the number of financial institutions, improve the level of financial services, and make use of modern financial means, such as digital finance. Meanwhile, we must also consider how to improve farmers’ financial acceptance and reduce their self-exclusion. This study’s results show that we must strengthen farmers’ personality education, reduce the negative aspects of their personality traits, and improve the positive aspects of their personality traits.

## Limitations and future research direction

The conclusion of this Manuscript shows that price exclusion is not the main factor affecting farmers’ credit exclusion. This does not mean that farmers do not care about loan interest rates but that farmers can enjoy low-interest or interest-free loans under China’s national rural revitalization strategy, which does not impose a real market interest rate. In follow-up research, the author will investigate financial institutions, and study the impact of farmers’ personality traits on price exclusion under the condition of determining market interest rates.

## Data availability statement

The raw data supporting the conclusions of this article will be made available by the authors, without undue reservation.

## Author contributions

YT performed the conceptualization, methodology, visualization, and wrote the original draft. YF performed the conceptualization, methodology, validation, and wrote the original draft. GH was involved in the conceptualization, methodology, validation, and writing—review and editing. YT, YF, and GH collected the data, contributed to the article, and approved the submitted article.
